# *In Vitro* Evaluation of Scaffolds for the Delivery of Mesenchymal Stem Cells to Wounds

**DOI:** 10.1155/2015/108571

**Published:** 2015-10-04

**Authors:** Elizabeth A. Wahl, Fernando A. Fierro, Thomas R. Peavy, Ursula Hopfner, Julian F. Dye, Hans-Günther Machens, José T. Egaña, Thilo L. Schenck

**Affiliations:** ^1^Department of Plastic Surgery and Hand Surgery, University Hospital Klinikum rechts der Isar, Technical University Munich, 81675 Munich, Germany; ^2^Institute for Regenerative Cures, UC Davis, Sacramento, CA 95817, USA; ^3^Department of Biological Sciences, California State University, Sacramento, CA 95819, USA; ^4^Institute of Biomedical Engineering, UCL, The Royal Institution, London W1S 4BS, UK; ^5^FONDAP Center for Genome Regulation, Faculty of Sciences, University of Chile, 7800024 Santiago, Chile

## Abstract

Mesenchymal stem cells (MSCs) have been shown to improve tissue regeneration in several preclinical and clinical trials. These cells have been used in combination with three-dimensional scaffolds as a promising approach in the field of regenerative medicine. We compare the behavior of human adipose-derived MSCs (AdMSCs) on four different biomaterials that are awaiting or have already received FDA approval to determine a suitable regenerative scaffold for delivering these cells to dermal wounds and increasing healing potential. AdMSCs were isolated, characterized, and seeded onto scaffolds based on chitosan, fibrin, bovine collagen, and decellularized porcine dermis. *In vitro* results demonstrated that the scaffolds strongly influence key parameters, such as seeding efficiency, cellular distribution, attachment, survival, metabolic activity, and paracrine release. Chick chorioallantoic membrane assays revealed that the scaffold composition similarly influences the angiogenic potential of AdMSCs *in vivo*. The wound healing potential of scaffolds increases by means of a synergistic relationship between AdMSCs and biomaterial resulting in the release of proangiogenic and cytokine factors, which is currently lacking when a scaffold alone is utilized. Furthermore, the methods used herein can be utilized to test other scaffold materials to increase their wound healing potential with AdMSCs.

## 1. Introduction

Mesenchymal stem cells (MSCs) have been shown to improve tissue regeneration* in vitro* and* in vivo*. Clinical data corroborates their beneficial regenerative effects in several organs and tissues, such as the heart, nerves, bone, and skin [1–4]. In order to administer MSCs to patients, cells have been introduced systemically and locally. While MSCs do have a homing capability to migrate to injured tissue, it has been claimed that after systemic administration only a fraction of the cells can migrate to the target tissue, while the majority of cells accumulate in the kidneys and lungs [5, 6]. In the case of local injections, a large number of these cells are required and while a substantial proportion of the cells remain in the area, another quantity is flushed out into the blood circulation [2, 7]. In an attempt to increase the retention rate of the cells, MSCs have been applied in association with biomaterials; for example, fibrin sprays and microbeads have been used for chronic skin wounds [8, 9], while meshes and three-dimensional scaffolds have been used to treat ischemic heart tissue [10] and diabetic ischemic ulcers [11].

Engrafted MSCs can release a series of cytokines and growth factors by interacting with local tissue to enhance repair and regeneration [5, 12]. Recent studies indicate that MSCs modulate the regenerative microenvironment by means of a controlled release of several paracrine factors related to key processes, such as angiogenesis, cell homing, immunomodulation, tissue remodeling, and fibrosis [13–15]. Thus, MSCs may impact regeneration primarily by releasing paracrine factors necessary for wound healing [16–18] rather than tissue replacement.

While MSCs have been found to exist in nearly every adult tissue [19–24], the proliferation rate of MSCs derived from adipose tissue (AdMSCs) is not affected by donor age [25–27], making it possible to use them in an autologous manner in elderly patients in regenerative medicine. A high quantity of MSCs can be obtained from a small amount of fat tissue (at least 1 × 10^6^ AdMSCs can be obtained from 200 mL of lipoaspirates) with more than 90% viability and virtually no harm to the donor [28, 29]. Furthermore, as vasculature is believed to be rich in MSCs, it is not surprising that a large quantity of AdMSCs can be isolated from a small amount of adipose tissue, which is highly vascularized [30, 31].

Several studies have shown the immunosuppressive properties of AdMSCs, which has allowed for xenogeneic transplantation into immunocompetent recipients for various disease models evidencing significant improvement without suppressing the immune system [31, 32]. Furthermore, clinical and preclinical studies have determined that allogeneic transplants of AdMSCs do not usually result in graft-versus-host disease (GvHD). These transplants have been used to treat GvHD after hematopoietic stem cell transplantation [32–34].

The positive effects of the use of MSCs are well established for various tissues; however, several regulatory and practical issues make chronic ulcers an attractive target for the clinical use of MSCs. More importantly, chronic ulcers remain an eminent clinical problem negatively impacting patients' quality of life and simultaneously representing a substantial expenditure for the healthcare system. In the US, these problems affect more than 8 million people with annual costs of around $20 billion [35]. With an aging population and the likelihood that the majority of the healthcare costs will come from patients over 65, the costs are almost certain to increase [36].

Several studies have proposed the combined use of scaffolds for dermal regeneration with stem cells for the treatment of chronic skin ulcers. In those studies, it has been shown that after seeding cells are able to survive in scaffolds, releasing several bioactive molecules that enhance skin regeneration* in vivo* [7, 37–39]. Although the results of preclinical trials are robust, several issues have to be clarified and optimized before clinical translation. In the case of chronic wounds, the cells must produce optimum amounts of paracrine factors in order to achieve the quantity necessary for healing. The addition of AdMSCs to the scaffold should support the healing process by creating a proregenerative microenvironment in the wound area. The key issue of determining the best combination of cells with a biomaterial and the development of an optimized composite material with increased regenerative capacity remains to be addressed.

Scaffolds alone are currently being used to treat chronic wounds in clinics and are composed of a variety of materials. In this study, we chose three scaffolds that are currently being used in clinics and one that is under development, all comprised of different biomaterials, to incorporate AdMSCs. BioPiel is a film-like scaffold derived from crustacean chitosan. Smart Matrix, currently under development, consists of a fibrin-alginate composite. Integra Dermal Regenerative Template (DRT) is a bilayer scaffold composed of type I bovine collagen and chondroitin-6-sulfate with a thin silicon layer and Strattice is derived from decellularized porcine dermis.

In this study, we analyzed and compared the behavior of AdMSCs in four distinct scaffolds, which were chosen because of their differences in the construction, material, and protein composition. The seeding efficiency, cellular distribution, attachment, survival, metabolic activity, and paracrine release of the seeded cells were analyzed* in vitro* as were the angiogenic effects* in vivo*.

## 2. Materials and Methods

### 2.1. Cell Isolation and Culture

Adipose tissue was derived from lipoaspirates obtained from donors who had given informed consent to participate in the study. The aspirated fraction was added to 50 mL Falcon tubes with an equal volume of 0.3 U/mL collagenase A (Roche, Basel, Switzerland) and incubated for 30 min at 37°C. After centrifugation, the resulting stromal vascular fraction was plated under standard conditions in Dulbecco's Modified Eagle's Medium with 4.0 mg glucose/L, stable glutamine, phenol red (DMEM; Biochrom, Berlin, Germany), supplemented with 10% fetal calf serum (FCS; PAA, Pasching, Austria), and 1% penicillin/streptomycin (P/S; Biochrom) under standard cell culture conditions (37°C, 5% CO_2_) and medium was changed every 3-4 days. In all experimental settings, cells from passage 3 were used with three donors (*N* = 3) and performed in triplicate (*n* = 3).

### 2.2. Cell Characterization

For analysis of cell surface markers by flow cytometry, AdMSCs were detached from the culture flasks with trypsin-EDTA solution (Biochrom), rinsed with phosphate buffered saline (PBS; Biochrom) and incubated for 45 min with Phycoerythrin- (PE-) conjugated antibodies raised against CD45, CD73, CD90, CD105, and CD146 at 4°C (1 : 100 dilution) (*N* = 3, *n* = 3). As isotype controls, IgG-PE was used (all antibodies from BD Biosciences, San Jose, CA). Samples were examined with a Cytomics FC500 (Beckman Coulter, Brea, CA).

To test the osteogenic differentiation potential of the AdMSCs, 80–90% confluent cells were cultured for 18 d in either control medium (alpha-MEM (Biochrom) + 10% FCS and 1% P/S) or osteogenic medium (hMSC osteogenic differentiation BulletKit, Lonza, Basel, Switzerland) in 6-well plates with a medium change every 3-4 d. Then, cells were fixed with 10% v/v formalin solution for 15 min, rinsed with PBS, stained with 0.5% w/v Alizarin Red S indicator (Ricca Chemicals Company, Arlington, TX) 30 min with gentle shaking, washed 3 times with PBS, and imaged for calcium deposition.

To test adipogenic differentiation of AdMSCs, cells were seeded in 6-well plates to 80–90% confluence. Medium was changed to either control medium (alpha-MEM + 10% FCS and 1% P/S) or adipogenic induction medium (hMSC adipogenic differentiation BulletKit, Lonza). For Oil Red O staining, cells were fixed after 14 d with 10% v/v formalin solution, rinsed with PBS, and stained with Oil Red O (Electron Microscopy Sciences, Hatfield, PA), and adipocytes were imaged (Nikon Eclipse TS100 Inverted Microscope).

Chondrogenic differentiation potential was carried out with three-dimensional pellet cultures in 15 mL polypropylene conical tubes. The initial pellets contained 2.5 × 10^5^ cells and were cultivated for 21 d in either control medium or chondrogenic induction medium (hMSC chondrogenic differentiation BulletKit, Lonza) supplemented with TGF Beta 3 (Lonza). After collection, pellets were rinsed with PBS and fixed in formalin. Pellets were sectioned (5 *μ*m) in paraffin and stained with Alcian Blue to visualize acetic mucins and acid mucosubstances and counterstained with Nuclear Fast Red (both from Sigma-Aldrich, St. Louis, MO, USA) before imaging. All stainings were carried out with *N* = 3 and *n* = 3.

### 2.3. Scaffolds

Four scaffolds, based on different biomaterials, were tested in this study. Here we compared BioPiel (chitosan film), Smart Matrix (fibrin matrix), Integra DRT (collagen-glycosaminoglycan matrix), and Strattice (decellularized dermis). BioPiel (Recalcine, Santiago, Chile) is a commercially available wound dressing with hemostatic and bacteriostatic properties composed of chitosan. Smart Matrix (RAFT, Northwood, Middlesex, UK) is a porous cross-linked fibrin-alginate composite biomaterial and is not yet commercially available. Integra DRT (Integra Life Sciences, Plainsboro, NJ, USA) is a commonly used, FDA approved, biodegradable porous scaffold based on bovine type I collagen fibers that are cross-linked by glycosaminoglycans (GAG) with a protective silicon layer. Strattice (LifeCell Corporation, Branchburg, NJ, USA) is an FDA approved porcine decellularized dermal matrix. In all experiments, 6 mm (in diameter) discs, as created with a biopsy punch, were used.

### 2.4. Fluid Capacity of the Scaffolds

In order to determine the maximum seeding volume, the fluid uptake of each scaffold was determined. Dried matrices were placed in DMEM and their fluid capacity was calculated (*n* = 8) [41]:

(1) $$\begin{array}{c} \text{Hydrophilicity}=\frac{\text{wet weight}-\text{dry weight}}{\text{dry weight}}. \end{array}$$

### 2.5. Structural Analysis of the Scaffolds

The micro- and macrostructures of the scaffolds were analyzed by scanning electron microscopy (SEM) and optical microscopy, respectively. Scaffolds were dehydrated with graded ethanol, air-dried, and sputter-coated with gold for 80 sec at 40 mA (Sputter Coating Device SCD 005, Bal-Tec AG, Liechtenstein). Analysis was performed at 5 kV accelerating voltage in a scanning electron microscope (Jeol JSM-5400, Japan). For macroanalysis, scaffolds were imaged using a stereoscope (Zeiss, Jena, Germany) from the side and top view.

### 2.6. Cell Seeding of Scaffolds

Scaffolds were placed in 24-well plates and 1.8 × 10^5^ AdMSCs were seeded dropwise with defined volumes of DMEM, supplemented with 10% FCS and 1% P/S according to the fluid capacity of the scaffold (Chitosan film: 35 *μ*L, Fibrin matrix: 25 *μ*L, Collagen-GAG matrix: 40 *μ*L and, Decellularized dermis: 22 *μ*L). AdMSCs were suspended in DMEM and seeded dropwise directly onto the scaffold. After 1 h, 1 mL of additional medium was added to the scaffolds, which were further cultured under standard conditions.

### 2.7. Cell Seeding Efficiency on Scaffolds

The percentage of cells incorporated into the scaffolds (*n* = 3, *N* = 4) was quantified by counting the cells attached to the culture dish one hour after seeding, that is, cells that did not attach to the scaffold. The scaffolds were removed and the remaining cells were detached from the well plates with trypsin-EDTA solution and counted in a Neubauer chamber. Seeding efficiency was calculated as the percentage of cells in the scaffold from the total number of seeded cells.

### 2.8. Cellular Distribution throughout Scaffolds

AdMSC-containing scaffolds were rinsed with PBS, fixed (3.7% paraformaldehyde, 0.1% Triton in PBS) on ice for 30 min, and blocked in 2% BSA in PBS at 4°C overnight (*N* = 3, *n* = 3). Scaffolds were then incubated in a blocking solution containing 2 U/mL Texas Red-X Phalloidin (Life Technologies, Grand Island, NY) to stain polymerized actin and 3.5 *μ*M To-Pro-3 (Life Technologies) to stain DNA. After washing 4 times with PBS (10 min each), scaffolds were dried with sterile gauze, mounted in Vectashield Mounting Medium (Vector Labs, Burlingame, CA) on glass bottom culture dishes (MatTek Corp., Ashland, MA), and imaged using an Olympus Fluoview FV10i confocal microscope (Olympus, Tokyo, Japan). Chitosan films were z-section imaged from top to bottom with the drop side facing down on the glass bottom, in 4 independent locations (one center and 3 periphery locations). As the fibrin matrix, collagen-GAG matrix, and decellularized dermis are too thick for visualization by confocal microscopy from top to bottom, they were sectioned using a razor blade and rotated onto their sides in order to generate z-section images from cross sections. Image analysis to assess cell morphology, number, and distribution was performed using Olympus FV10-ASW software (Olympus).

### 2.9. Metabolic Activity and Cytotoxicity in the Scaffold

On days 1, 3, 7, and 14 after seeding, the metabolic activity of the seeded cells was evaluated by precipitation of tetrazolium salt (WST-1). Cellular death was measured by the release of lactate dehydrogenase (LDH) from the cells on days 1, 3, and 7 (both from Roche, Mannheim, Germany) (*n* = 3, *N* = 3). As the medium needed to be changed after 7 d, the total LDH activity could not be measured over a 14 d period. Seeded scaffolds were incubated in DMEM and WST-1 solution (1 : 10 ratio) for 1 h. The absorbance of the resulting formazan dye was measured at 450 nm with a reference wavelength of 620 nm. For the measurement of LDH, supernatants were harvested from the same scaffolds used for WST-1 assay and the analysis was performed according to the manufacturer's instructions. In short, the absorbance was measured at 490 nm and a reference wavelength of 620nm with controls including medium alone (background), cells in well plates without scaffolds (spontaneous LDH release), and cells in well plates without scaffolds with Triton X-100 in the medium (maximum LDH release). The resulting value was then calculated with the equation: cytotoxicity (%) = (experimental value – spontaneous LDH release)/(maximum LDH release – spontaneous LDH release) × 100.

### 2.10. Characterization of Secretion Profile

Supernatants were collected from AdMSC seeded on scaffolds or tissue culture plastic (*n* = 3, *N* = 3; 1.8 × 10^5^ cells/scaffold) after 48 h under standard cell culture conditions, shock frozen with liquid nitrogen, and stored at −80°C until analysis. Human Cytokine and Angiogenesis Array Kits (R&D Systems, Abingdon OX, UK) were used to characterize the release of multiple cytokines and angiogenesis related proteins, respectively. Membranes were imaged using a Peqlab Fusion FX7 chemiluminescence system (Erlangen, Germany) and the spot intensity was quantified with ImageJ software [42] using the MicroArray Profile plugin (OptiNav, Inc.). Scaffolds without cells served as controls.

### 2.11. Hypoxia-Inducible Factor-1*α* Expression

Seeded scaffolds (*n* = 3, *N* = 3) were incubated in standard (21% O_2_, 5% CO_2_) or hypoxic conditions (1% O_2_, 5% CO_2_) and collected at 4, 8, and 16 h. Then scaffolds were washed two times with sterile PBS. Three scaffolds from each time point, oxygen condition, and type were briefly sonicated in 500 *μ*L lysis buffer; the HIF-1*α* expression was analyzed using a Human Total HIF-1*α* ELISA kit (R&D Systems), and the optical density was measured at 450 nm using a Mithras LB 940 Microplate Reader. The total protein concentration was determined using a Pierce BCA Protein Assay Kit (Thermo Scientific, Rockford, IL) and the absorbance was measured at 560 nm.

### 2.12. Chicken Chorioallantoic Membrane (CAM) Assay

Research grade fertilized eggs (SPF, Valo Biomedia GmbH, Osterholz-Scharmbeck, Germany) were placed on a rotating egg tray for 3 days after fertilization at 37°C and 60–70% humidity. On day 3, a small window was made in the shell under aseptic conditions and the contents of the egg were gently placed into a 200 mL plastic dish. The dish was further placed into a petri dish with 50 mL of distilled water, 1% P/S, and 1% partricin and incubated at 70–80% humidity to prevent drying of the membrane. On day 10, autoclaved filter paper punches (5 mm) were added to the CAM directly followed by 10 *μ*L of conditioned media collected from serum-free cell seeded scaffolds after 48 h in culture, DMEM, PBS, or 20 ng of VEGF, which was reapplied daily for 3 days [43]. The applied filter paper punches were imaged daily using a Canon EOS 20D digital SLR camera with a Canon EF 50 mm f/1.8 II Standard AutoFocus Lens. Samples were quantified (*n* = 3, *N* = 6) by being given arbitrary values based on the distribution and density of CAM vessels around the filter paper punch [40].

### 2.13. Statistical Analysis

Results were analyzed with GraphPad Prism version 6.0e for Mac OSX (GraphPad Software, San Diego, CA USA) and are shown as mean ± standard deviation. Significant differences between sample groups were determined by analysis of variance (ANOVA) with a Bonferroni posttest where *p* < 0.05 was considered statistically significant.

## 3. Results

**Figure 1:**
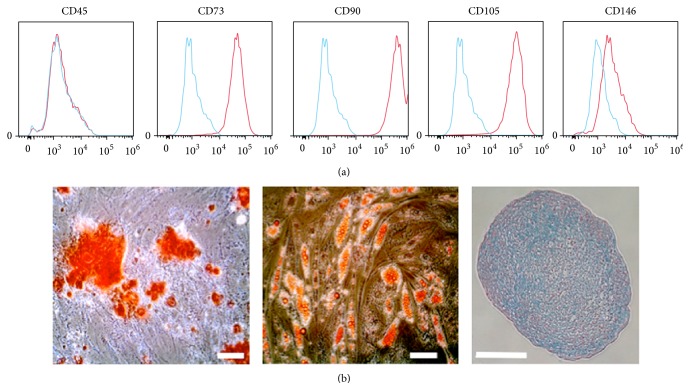
AdMSC characterization. The immune phenotype of the cells was evaluated by labeling cells with Phycoerythrin-conjugated antibodies for flow cytometry. The blue histogram indicates the isotype control (a). In order to evaluate their differentiation potential, AdMSCs were cultured with control (not shown), osteogenic, adipogenic, or chondrogenic medium (b). Calcium precipitation as a result of osteogenic differentiation was confirmed by Alizarin Red S staining (left panel), triglyceride-containing vacuoles emerging from adipogenic differentiation were stained used Oil Red O (middle panel), and cartilaginous glycosaminoglycans and mucins were stained using Alcian Blue with a Nuclear Fast Red nucleic cross stain. Staining was performed after 21 d in culture. Scale bars represent 100 *μ*m. *n* = 3, *N* = 3.

Mesenchymal stem cells were isolated from human adipose tissue and characterized in terms of their immune phenotypes and differentiation potential. Fluorescence-activated cell sorting analysis showed that AdMSCs do not express pan-hematopoietic marker CD45 but are positive for CD73, CD90, and CD105 (Figure 1(a)). Interestingly, AdMSCs expressed very low levels of the pericyte marker CD146. Moreover, the cells showed a strong differentiation potential towards osteoblasts, adipocytes, and chondrocytes after culturing in their respective differentiation conditions (Figure 1(b)). Calcium deposits were stained with Alizarin Red S for AdMSCs exposed to osteoblast differentiation medium. Lipid vacuoles from adipogenic differentiation were stained with Oil Red O. Chondrogenic pellets were stained with Alcian Blue to show chondrocyte growth.

**Figure 2:**
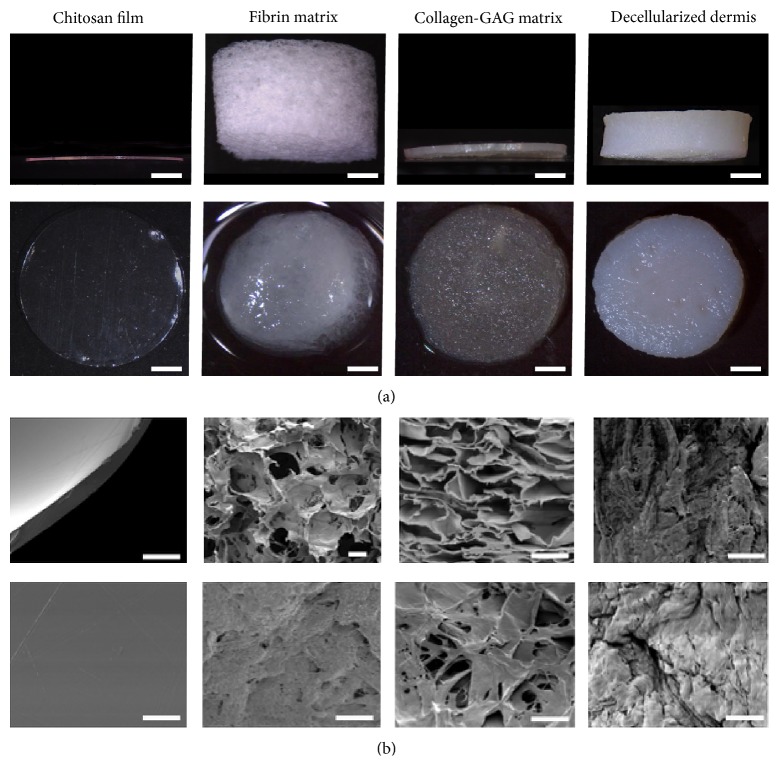
Scaffold characterization. The thickness and general structure of the scaffolds (6 mm in diameter) were analyzed macroscopically from the side (dry, top row) and the top (wet, bottom row). The thickness of the scaffolds remains the same except for the fibrin matrix, which collapses into a mesh of fibers. Scale bars represent 1 mm (a). The pore structure and texture of the scaffolds were analyzed by SEM micrographs from the transverse sections (top row) and top view (bottom row). Note the complete absence of pores in the chitosan film in comparison to the other scaffolds. Scale bars represent 100 *μ*m (b).

In this work, four different scaffolds were compared for their usability with AdMSCs in dermal regeneration (Table 1). We evaluated and compared the macro- and microstructure of the four scaffolds, observing important differences (Figure 2). The dry thickness of the scaffold varies from a minimum of 0.12 mm for chitosan films to 3.8 mm for fibrin matrices (Figure 2(a)). When wet, the structure of the fibrin matrices collapses to a fibrous mesh decreasing the measurable thickness. The decellularized dermis had the thickest structure at 1.5 mm, while the collagen-GAG matrix was 0.20 mm thick (Table 1). Compared to the other scaffolds, the chitosan has a film-like appearance, while the fibrin and collagen-GAG present a more mesh-like structure and exhibited high porosity throughout the scaffolds. The decellularized dermis exhibited much tighter pores and the chitosan did not have any visible porosity (Figure 2(b)).

Scaffold porosity and the degree to which the pores are interconnected determine the loading capacity of the scaffolds. After AdMSCs were seeded and allowed to attach to the scaffold for one hour the seeding efficiency was evaluated. A seeding efficiency of almost 90% was observed in the fibrin matrix (88.6 ± 2.9), collagen-GAG matrices (86.5 ± 3.8), and the decellularized dermis (89.2 ± 3.8), which was significantly higher than the chitosan films (60.1% ± 5.9%) (*p* < 0.05).

**Figure 3:**
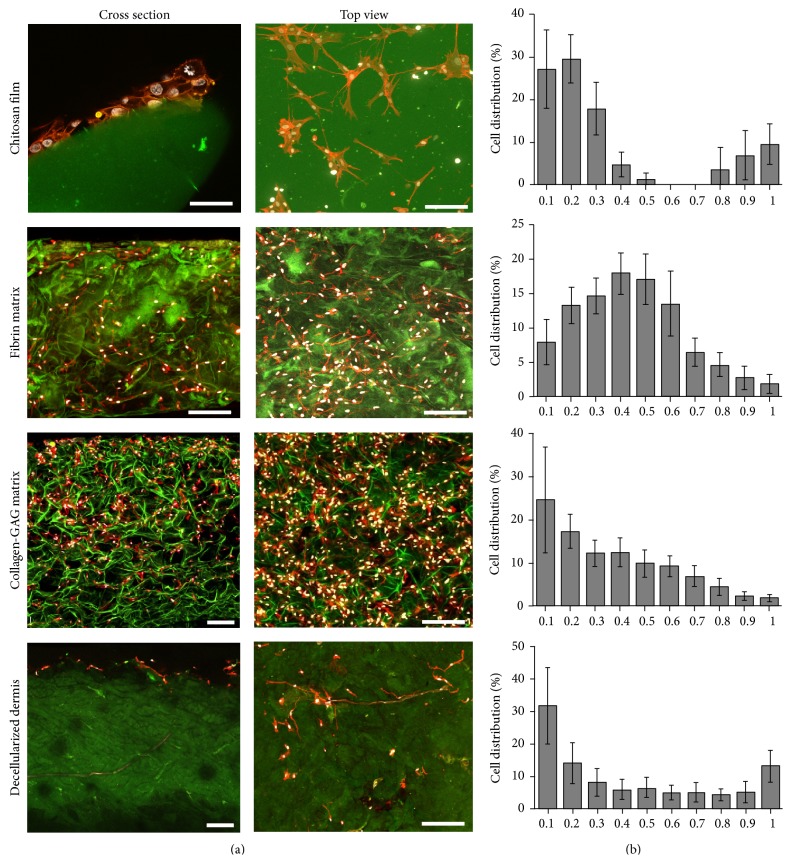
Cellular distribution and attachment of cells on scaffolds. After seeding, the distribution and attachment of the cells was evaluated by LSM. In all cases, except for the decellularized dermis, AdMSCs (To-Pro-3 (white)/phalloidin (red)) adhered to the scaffold (green autofluorescence) showing a fibroblast like morphology. As can be seen in the first column, cells formed a layer over chitosan films while the others cells were able to migrate further into the scaffold. Cross section (right) and top view (left) of scaffolds. Scale bar represents 150 *μ*m (a). Quantification of the cellular densities after 1 d throughout sections, ranging from the top (0.1) to the bottom (1) of the scaffolds as seen in confocal imaging (b). *n* = 3, *N* = 3.

Differences in the mechanical properties should influence the cell behavior when seeded. For that, a detailed view into the interaction and distribution of the seeded cells in the scaffold was obtained by confocal microscopy. Except for the decellularized dermis, the AdMSCs were highly attached to the material, showing fibroblastic morphology, creating a complex tridimensional arrangement between the cells and the scaffold (Figure 3(a)). The images were analyzed to give quantitative, spatial information on the cellular distribution throughout the scaffold (Figure 3(b)). The AdMSCs formed a layer on the seeding surface of chitosan films, showing almost no cells in the core. In the case of the fibrin matrix, cells were observed throughout the scaffold with a tendency to accumulate at the inner core of the material. Cells seeded on collagen-GAG matrices also showed a different distribution pattern creating a cell gradient from the seeding side to the bottom. In the decellularized dermis, AdMSCs were more concentrated on the seeding side while migration through the scaffold was limited.

**Figure 4:**
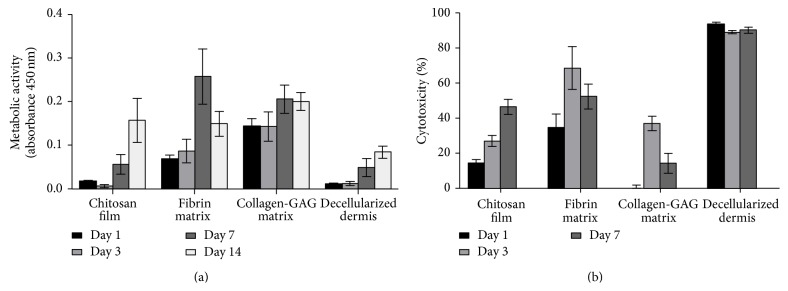
Cellular survival within the scaffold. The metabolic activity (WST-1) and cellular death (LDH) were measured and compared after seeding. Results show that the metabolic activity of the cells increased over time, indicating that the cells were able to proliferate within the scaffolds (a). The LDH released by the cells was measured as an indicator of cellular death. The highest mortality was observed after only 1 d in the decellularized dermis indicating a poor biocompatibility with the AdMSCs (b). *n* = 3, *N* = 3.

The distribution of the AdMSCs is an important indicator for biocompatibility with the different scaffold materials. However, secretion activity relies on cell survival beyond the initial seeding, which was measured by means of their metabolic activity. Interestingly, there was no correlation between the metabolic activity and seeding efficiency. Twenty-four hours after seeding, the formation of formazan blue, as an indicator of metabolic activity, was the highest in fibrin and collagen-GAG matrices, while AdMSCs seeded on the chitosan film and decellularized dermis showed comparable values initially (Figure 4(a)). In order to evaluate if these differences were due to increases in cellular death, lactate dehydrogenase (LDH) activity was measured from the supernatants. It can be seen that the decellularized dermis had a high rate of cytotoxicity (almost 100%), even after only one day in culture, whereas the collagen-GAG matrix had virtually no cytotoxic effect (Figure 4(b)).

The long-term viability of the AdMSCs seeded on the scaffolds was measured and compared at further time points after seeding. Results show that while the chitosan film and decellularized dermis have comparable metabolic activity through day 7, 14 days after seeding the chitosan film exhibited similar results to the fibrin and collagen-GAG matrices (Figure 4(a)). The collagen-GAG matrix showed steady metabolic activity throughout the 14 days, while the fibrin matrix showed an increase in activity through day 7 after which the activity decreased at day 14. Cellular death results showed a general increase in cytotoxicity as the metabolic activity of the cells increased, except for in day 7 of the fibrin and collagen-GAG matrix where the metabolic activity peaked. The highest percentage of cytotoxicity was seen in the decellularized dermis being close to 100% (Figure 4(b)).

**Figure 5:**
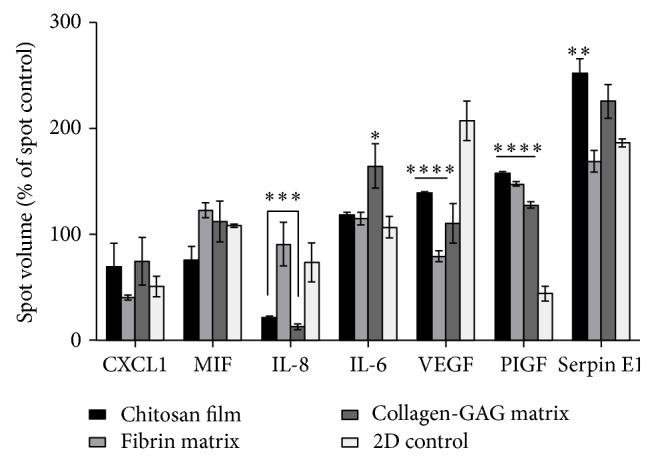
Secretion profile of AdMSC seeded scaffolds. Human cytokine and angiogenesis arrays were utilized in order to analyze supernatants after a 48 h incubation to detect if there is an effect of the scaffolds interaction with the cells on paracrine factor release. Decellularized dermis scaffolds were excluded as previous data revealed that it did not provide a compatible environment for the cells to migrate and flourish. Chitosan films and collagen-GAG matrices show a decrease in expression of IL-8 in comparison to fibrin matrices, which is similar to two-dimensional conditions. Collagen-GAG matrices had a significant release of IL-6, while chitosan films had an increase of Serpin E1 release over all other conditions. There are significant differences in release of PIGF and VEGF from all scaffolds in comparison to two-dimensional cultures. ^*∗*^ *p* < 0.05; ^*∗∗*^ *p* < 0.01; ^*∗∗∗*^ *p* < 0.001; ^*∗∗∗∗*^ *p* < 0.0001 when compared to 2D control. *n* = 3, *N* = 3.

The differences detected between scaffolds in relation to the behavior of AdMSCs lead to the conclusion that depending on the physical and chemical conditions, the factors secreted from the AdMSCs can also vary considerably. Here, the secretion of 91 different angiogenic, cytokine, and chemokine factors were analyzed to obtain a characteristic secretion profile for each scaffold. Among the detected factors, the most prevalent one was macrophage migration inhibitory factor (MIF), plasminogen activator inhibitor 1 (Serpin E1), interleukin 6 (IL-6), interleukin 8 (IL-8), chemokine (C-X-C motif) ligand 1 (CXCL1), placental growth factor (PlGF), and vascular endothelial growth factor (VEGF) (Figure 5). Due to the low viability of the cells observed after seeding, decellularized dermis scaffolds were excluded for this assay.

Compared to AdMSCs seeded directly onto tissue culture plastic, the scaffold condition itself significantly induces the release of PlGF while it reduces the release of VEGF (*p* < 0.05). Compared among the scaffolds, we observed that the release of angiogenesis inducing IL-8 was similar between fibrin matrices and two-dimensional cultures, while chitosan films and collagen-GAG matrices show a dramatic decrease (*p* < 0.05). The release of inflammation regulating IL-6 was elevated in supernatants from cells seeded on collagen-GAG matrices while chitosan films showed the highest expression of Serpin E1. MIF and CXCL1 did not show any significant differences between scaffolds or control conditions.

A major problem in wound healing is the limited oxygen concentrations inhibiting healing. We used hypoxia inducible factor-1*α* (HIF-1*α*) to investigate if the scaffolds inhibit the cells from gaining access to oxygen levels. At time points 4, 8, and 16 h no noticeable traces of HIF-1*α* could be detected (data not shown). We concluded that all scaffolds allow proper gas exchange with the environment.

**Figure 6:**
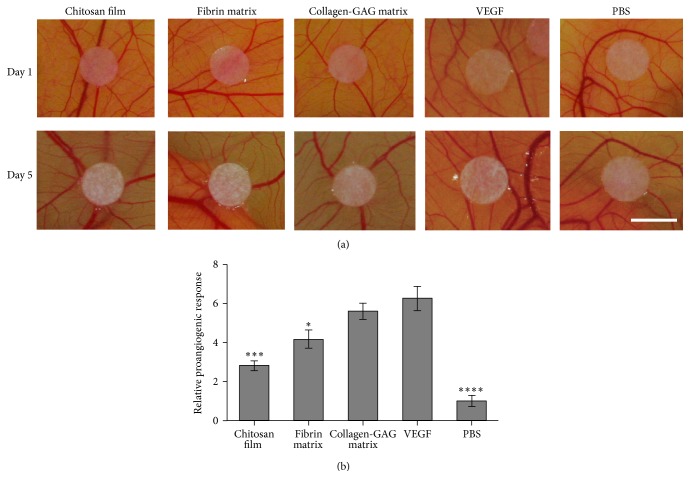
Chicken chorioallantoic membrane* in vivo* analysis. Autoclaved filter paper punches with conditioned medium from scaffolds after 48 h in culture were observed over a five-day period for neovascularization of the CAM. Note the increase in small vessel convergence from the scaffold, specifically in VEGF, collagen-GAG, and fibrin matrices (a). Samples exposed to conditioned medium without cells are not pictured, as they did not differ from the negative control (PBS). Growth was analyzed from 6 replicates per treatment based on an arbitrary scoring system dealing with new small vessel formation and the behavior of existing vessels as observed daily according to [40]. Briefly, a value was assigned for each 5 d ranging from (0) unchanged to slight changes in density and convergence towards filter paper punch (1) and further increases in density and convergence (up to 5) (b). 5 mm scale bar. ^*∗*^ *p* < 0.05, ^*∗∗∗*^ *p* < 0.001, ^*∗∗∗*^ *p* < 0.0001 when compared to VEGF positive control.

Finally, we evaluated the biological effects of conditioned medium in an* in vivo* CAM assay model. The chicken chorioallantoic membrane assay is an established method to monitor* de novo* vessel formation. Here, conditioned medium was pipetted onto autoclaved filter paper punches in order to determine if there was an enhanced effect from the factors secreted from the AdMSCs that was due to the composition of the scaffold or if the scaffold alone had any angiogenic potential. In order to minimize irritation to the CAM and avoid affecting the result, the scaffolds themselves were not utilized. In the positive control (VEGF), large existing vessels showed a tendency to move toward the filter paper, while this was not evident in the samples exposed to the AdMSC conditioned medium, suggesting that the supernatant of the cells was not as proangiogenic as pure VEGF (Figure 6(a)). Nevertheless, as seen in the* in vitro* data, the highest instance of neovascularization in small vessel convergence and growth occurred with medium that was obtained from collagen-GAG matrices followed by fibrin matrices and, finally, chitosan films (Figure 6). The quantification is based on arbitrary points given for* de novo* small vessel formation up to reorganization of existing vessels (Figure 6(b)) [40]. These results suggest that the composition of the scaffold has a direct effect on the angiogenic factors released from the AdMSCs. No significant differences appeared between PBS, conditioned medium without cells, and DMEM alone (data not shown for the medium exposed samples).

## 4. Discussion

Although various stem cell populations have been suggested for therapeutic use, MSCs are particularly attractive as they are well discerned and ongoing clinical trials have shown promising results in wounded tissue [4, 44–46]. Furthermore, there is great potential for using AdMSCs in regenerative medicine [1]. They are easy to isolate, are accessible with minimally invasive procedures, and contain a high number of cells within a small amount of tissue, and the age of the donor does not affect their proliferation rate or differentiation potential [26, 27], making them ideal for clinical procedures.

For clinical application, administration, and effectiveness are key factors in describing the efficacy of a given treatment. In the case of AdMSCs, this encompasses the viability of the cells under the given conditions and their ability to release beneficial growth factors to the damaged tissue. To minimize migration, which reduces the utility of the method, dermal scaffolds were used. The scaffolds examined here were of particular interest as they are currently in use or being tested for use in clinics, although their healing effectiveness to date has been subpar due to slow tissue revascularization. Furthermore, the scaffolds chosen were substantially different in structure (Figure 2) and composition (Table 1). The viability, migration, and growth factor release, especially of the angiogenic growth factors, were of particular interest in this study.

After analyzing the AdMSCs (Figure 1) and scaffolds (Figure 2) for individual characteristics, the distribution and attachment of the seeded cells was analyzed and compared. As expected, AdMSCs adhered to all of the scaffolds but due to their composition and properties, their distribution varied greatly.

### 4.1. Chitosan Films

Consistent with the lack of porosity detected in chitosan films (Figure 2(b)), the AdMSCs created a single layer on the seeding side with virtually no penetration into the material (Figure 3). A level of porosity must be available in order for cells to be able to penetrate the scaffold and form a network for cells to communicate without overcrowding. Other chitosan derived scaffolds contain artificial pores in order to facilitate cell migration [47, 48]. The seeding side is critical for chitosan-based scaffolds to generate either a superficial cell layer or to create an AdMSC interface between the scaffold and the wound bed. As there is only a layer of cells, these may migrate out of the scaffold soon after transplantation and the effects of the AdMSCs on the wound bed may be beneficial for a short time in order to start a pathway towards healing. While metabolic activity increased over time, an overcrowding of the cells could limit the potential of the AdMSCs to release healing factors. The antimicrobial properties of chitosan makes it a beneficial treatment for superficial wounds and burns, to minimize scarring, decrease pain sensation, and reduce inflammation [49].

### 4.2. Collagen-GAG and Fibrin Matrices

In contrast to the chitosan film, the AdMSCs seeded onto the fibrin and collagen-GAG matrices showed better penetration into the material (Figure 3), with distribution in fibrin matrices peaking at the center of the scaffold. This effect may be due to the apparent unevenness of the porosity at the center of the fibrin matrix, showing a larger pore structure on the top and bottom of the scaffold, in comparison to the center, which might inhibit the cells from migrating throughout the scaffold (Figure 2). MSCs have been shown to possess a strong attachment to fibrin by way of small binding domains with the cell membrane, something not found with other cell types [50]. The cellular gradient observed in collagen-GAG matrices showed a higher concentration at the seeding side with a steady amount of migration throughout the scaffold. As collagen is the main component of the ECM, the cells were expected to be able to attach and distribute throughout the scaffold. Beyond their porosity, as both collagen and fibrin are dominant in the ECM, it is no surprise that they demonstrate a high cellular bond. Furthermore, AdMSCs isolated from lipoaspirates have previously shown a high affinity for binding to ECM proteins [51]. Beyond that, AdMSCs seeded in collagen-GAG matrices exhibited the highest level of metabolic activity and lowest level of cytotoxicity on day one.

The fibrin and collagen-GAG matrices showed the highest amount of cell migration of the four scaffolds, though at different distributions, which could be attributed to differences in cellular adhesion and migration triggered by the material itself [52, 53]. Throughout the observation, the cells seeded on the collagen-GAG matrix evidenced the steadiest rate of metabolic activity and the lowest rate of cellular death indicating the most compatible relationship between cells and biomaterial.

### 4.3. Decellularized Dermis

Although AdMSCs seeded onto the decellularized dermis were able to migrate through the material, a strong decrease in metabolic activity was seen soon after seeding, indicating that it may not provide adequate space for the cells to thrive or may even induce cell death (Figure 4). This might be particularly important for the decellularized dermis as it went through cell removal during preparation. The decellularized dermis utilized here, Strattice, has been used successfully as an internally placed scaffold for treatment of subcostal hernia repair [54] and breast reconstruction [55, 56]. Mirastschijski et al. have found that the decellularized dermis may be best suited for dermal wound beds that require a higher mechanical load than in those previously mentioned [57]. While residual porosity does facilitate some AdMSC migration, the high mortality rate would make this an unsuitable scaffold for a cell seeded dermal wound treatment. This high mortality rate may be due to residual chemicals from the decellularization process that cannot be easily washed away before cell seeding. However, the low cellular infiltration that we observed* in vitro* is in line with previous data showing similar results after subcutaneous implantation of the scaffold in a rat model [58].

Although the metabolic activity increased over time in the decellularized dermis, despite the initial rate of cellular death, of the four examined it seems to be the least compatible combination of the AdMSCs and biomaterial. This may be a result of cell overcrowding, due to tight porosity, and could limit the number of cells able to flourish. In addition, pore sizes in the decellularized dermis were not uniform enough in size for the cell-cell interaction necessary for the cells to thrive. Furthermore, the low metabolic activity observed over two weeks of seeding correlates with a high count of cell death only one day after seeding.

### 4.4. Scaffold-AdMSC Secretion Profile

During the first days after wounding, the release of paracrine factors is crucial for healing [59]. Independent of their differentiation capacity, MSCs have been shown to act as anti-inflammatory and immunoregulatory agents [59, 60], promote cell migration and proliferation and angiogenesis, and improve scarring [4]. The application of AdMSC seeded scaffolds to wounds could, therefore, be beneficial in all the three phases of wound healing: inflammation, proliferation, and tissue remodeling.

The physical and chemical conditions experienced by the cell can alter the cell behavior, in general, and the secretion profile, specifically. In addition, there can be further influences by exposure to biomolecules on the scaffolds, such as peptides and proteins, either artificially or intrinsically [61, 62]. Although the four scaffolds' chitosan is the only material that is not found in the human body, it has been employed successfully in wound healing treatments [49]. Surprisingly, the chitosan film released significantly higher levels of Serpin E1 than the control cells and fibrin matrices. Serpin E1 is known to regulate extracellular matrix (ECM) remodeling [63], which is why a high level of expression from the collagen-GAG matrices is expected (Figure 5).

VEGF and PlGF work together to induce angiogenesis, endothelial cell growth, and promote cell proliferation and migration. VEGF expression is dependent on PlGF while the PlGF/VEGF heterodimer induces pathological angiogenesis [64–66]. In general, the scaffolds had a significant effect in reducing VEGF and increasing PlGF expression relative to the two-dimensional culture (Figure 5). As little difference was found between the release of these factors from cells on each scaffold, this may imply that the scaffold itself upregulated the angiogenic potential of AdMSCs.

An increase in IL-6 may accelerate wound healing by increasing rates of angiogenesis and epithelial cell migration [18]. The scaffold composition did not seem to affect the release of this cytokine, except in the case of the collagen-GAG matrix where it was upregulated. IL-6 is known to induce collagen and GAG production [67] and a similar increase in IL-6 can be seen with primary human dermal fibroblasts seeded on a collagen-GAG matrix [68]. IL-6 also functions in pro- and anti-inflammatory situations and is a major regulator of acute phase reactions, which indicates a wound-like stimulation* in vitro*. IL-8 had a much lower expression rate in the chitosan film and collagen-GAG matrices. Fibrin is known to induce IL-8 expression in human umbilical vein endothelial cells (HUVECs) [69] and a relatively high expression of IL-8 was found in a previous study utilizing primary human dermal fibroblasts on the fibrin matrix [68]. IL-6 has been linked to angiogenesis by increasing VEGF expression [70], while IL-8 has been shown to upregulate VEGF in endothelial cells [71] and bone marrow derived MSCs [72] via signaling pathways.

A hypoxic environment creates cell stress and triggers AdMSCs to release angiogenic factors via an upregulation of HIF-1*α* [73]. No HIF-1*α* expression could be detected in cells seeded on scaffolds implying that the scaffolds themselves do not create a hypoxic environment. In the case of the chitosan scaffold, there was little cellular penetration creating a two-dimensional like environment. The pore sizes in both the fibrin and collagen-GAG matrix were likely connected and large enough for proper gas exchange. While most of the cells died quickly that were seeded on the decellularized dermis, it is hard to gauge if the scaffold itself would create a hypoxic environment.

The results suggest that the scaffold allows for proper gas exchange, which is most likely explained by the thickness of the scaffolds. Proper gas exchange should not be hindered in a scaffold less than 200 *μ*m [74]. In larger three-dimensional scaffolds, oxygen was depleted after 7 days [75]. As Strattice is 1.5 mm thick (Table 1) this could also pose a problem for the survival of the cells. The other three scaffolds should not be affected as the thickness after cell seeding is less than 200 *μ*m. Furthermore, MIF and VEGF are both regulated by HIF-1*α* [73]. Even though the control shows a higher release of VEGF, there were no significant differences between the scaffolds and controls with MIF. Therefore, there is little chance of the scaffolds creating a hypoxic environment.

The cells released factors into the medium that contributed to increased angiogenesis* in vivo* as tested in the well-established CAM assay. The CAM offers an exceptional model, as there is no immune system and the vascular networks are exposed. The conditioned medium from the collagen-GAG matrix showed no significant difference in small vessel convergence and growth from that of the VEGF positive control (Figure 6(a)). The medium from the scaffolds themselves did not differ from the observed vascular growth when using PBS, indicating that the synergistic effects of the AdMSCs with the scaffolds were the main component in the increased rates of angiogenesis. Interestingly, the high levels of VEGF and PIGF released from the chitosan film* in vitro* did not seem to have a strong effect here. As the cell seeded scaffolds were not used directly on the CAM, to prevent irritation, there could still be an effect from the other factors released by the cells on the chitosan film that inhibits vascular growth. This may also indicate inhibitory effects from the material of the chitosan film.

These results are remarkable as they show that scaffolds not only can be designed to harbor AdMSCs but also should be optimized to work synergistically with the cells in order to enhance the release of necessary and desirable factors to enhance wound healing by promoting angiogenesis, reducing healing time, and minimizing scar tissue.

## 5. Conclusions

In this work, a suitable delivery vehicle for AdMSCs to the wound that can secrete factors to facilitate healing was evaluated. AdMSCs in conjunction with the different scaffold types examined released angiogenic factors and chemokines necessary for wound healing. Although the decellularized dermis (Strattice) is used in clinical settings, its lack of porosity and the poor environment it creates for the AdMSCs do not make it an ideal candidate for a cell seeded, topically applied wound treatment. Cells seeded on the chitosan film secreted factors that are helpful in wound healing although the scaffold lacked the capability to let cells migrate throughout, leaving a crowded film of cells at the seeding side which could be lost upon transplantation. The ability for the scaffold to provide (i) an ideal environment for the cells to migrate, (ii) porosity that facilitates cell migration and crosstalk, and (iii) a biocompatible material are necessary to achieve proper healing* in vivo*. Through our investigative efforts, the collagen-GAG and fibrin matrices proved to have the best potential under the applied conditions as a platform for AdMSCs to enhance wound healing* in vitro*. The* in vivo* CAM data correlates with the* in vitro* data to further show the collagen-GAG and fibrin matrices are superior in working with the AdMSCs to promote angiogenesis and thus speed healing.

## Figures and Tables

**Table 1:** Comparison of scaffold properties. The general properties of the scaffolds show a broad variation in weight, size, fluid capacity, and price of material. As the fibrin matrix is currently not commercially available some information could not be divulged.

	Commercial name	Company	Price	FDA approval	Dry weight	Fluid capacity	Dry thickness
			[USD/cm²]		[mg/cm²]	[*μ*L/cm²]	(mm)
Chitosan film	**BioPiel**	Recalcine	10	Yes	4 ± 0.3	123 ± 14.8	0.12
Fibrin matrix	**Smart Matrix**	RAFT	—	N/A	12 ± 1.5	87 ± 9.0	3.8–1.8
Collagen-GAG matrix	**Integra DRT**	Integra Life Sciences	3	Yes	10 ± 1.4	143 ± 5.6	0.20
Decellularized dermis	**Strattice**	LifeCell	26	Yes	148 ± 2.9	77 ± 4.1	1.5
